# How oral probiotics affect the severity of an experimental model of progressive multiple sclerosis? Bringing commensal bacteria into the neurodegenerative process

**DOI:** 10.1080/19490976.2020.1813532

**Published:** 2020-09-08

**Authors:** Leyre Mestre, Francisco J. Carrillo-Salinas, Ana Feliú, Miriam Mecha, Graciela Alonso, Carmen Espejo, Laura Calvo-Barreiro, José L. Luque-García, Héctor Estevez, Luisa María Villar, Carmen Guaza

**Affiliations:** a Neuroimmunology Group, Functional and Systems Neurobiology Department, Instituto Cajal, CSIC, Madrid, Spain; b Red Española de Esclerosis Múltiple (REEM); c Tufts University School of Medicine, Boston, MA, USA; d Servei de Neurología-Neuroimmunología, Centre d’Esclerosi Múltiple de Catalunya, Vall d’Hebron Institut de Recerca, Hospital Universitari Vall d’Hebron, Barcelona, Spain; e Universitat Autònoma de Barcelona, Bellaterra, Spain; f Analytical Chemistry Department, Complutense University of Madrid, Madrid, Spain; g Immunology Department, Hospital Universitario Ramón y Cajal de Investigación Sanitaria (IRYCIS), Madrid, Spain

**Keywords:** Theiler’s virus model, probiotics, gut microbiota, neuroinflammation, multiple sclerosis, treg cells, breg cells

## Abstract

A growing number of studies support that the bidirectional interactions between the gut microbiota, the immune system and the CNS are relevant for the pathophysiology of MS. Several studies have reported alterations in the gut microbiome of MS patients. In addition, a variety of studies in animal models of MS have suggested that specific members of the gut commensal microbiota can exacerbate or ameliorate neuroinflammation. Probiotics represent oral nontoxic immunomodulatory agents that would exert benefits when using in combination with current MS therapy. Here we investigate the effect of Vivomixx on the gut microbiome and central and peripheral immune responses in a murine model of primary progressive MS. Vivomixx administration was associated with increased abundance of many taxa such as *Bacteroidetes, Actinobacteria, Tenericutes* and *TM7*. This was accompanied by a clear improvement of the motor disability of Theiler’s virus infected mice; in the CNS Vivomixx reduced microgliosis, astrogliosis and leukocyte infiltration. Notably, the presence of Breg cells (CD19^+^CD5^+^CD1d^high^) in the CNS was enhanced by Vivomixx, and while spinal cord gene expression of IL-1β and IL-6 was diminished, the probiotic promoted IL-10 gene expression. One of the most significant findings was the increased plasma levels of butyrate and acetate levels in TMEV-mice that received Vivomixx. Peripheral immunological changes were subtle but interestingly, the probiotic restricted IL-17 production by Th17-polarized CD4^+^ T-cells purified from the mesenteric lymph nodes of Theiler’s virus infected mice. Our data reinforce the beneficial effects of oral probiotics that would be coadjuvant treatments to current MS therapies.

## Introduction

Although the etiology of MS remains unknown, environmental and genetic risk factors are involved in the pathogenesis of the disease.^1^ Accumulating evidence indicate that microbiome gut dysbiosis may be implicated in the increased incidence of MS in developed countries.^2^ Specifically, bi-directional interactions between the gut microbiota, the intestinal barrier and the immune system appear to be relevant to the pathophysiology of MS.^3–5^ The different stages and subtypes of MS are influenced by environmental factors,^1^ particularly in the relapsing remitting MS forms (RRMS) with current successful therapies. Studies using the encephalomyelitis autoimmune experimental (EAE) model of MS described lower incidence in germ free (GF) or antibiotic treated mice than in specific pathogen free (SPF) mice,^6–8^ while recolonization restored the susceptibility to develop EAE.^3^ However, other studies reported a therapeutic role of probiotic administration in a variety of EAE models. On this matter, orally administration of *Bacteroides fragilis*,^9,10^ *Prevotella histicola,^11^* different species of *Lactobacillus (L. paracasei plus L. plantarum;*^12^ *L. Reuteri;^13^ L. helveticus*^14^), or multi-strain probiotics^15^ have demonstrated to improve EAE. In addition, probiotics supplementation to RRMS patients has been shown to modulate immunity by exerting anti-inflammatory effects.^16,17^

Right now, there is little information regarding the association between gut microbes and CNS inflammation/neurodegeneration in the primary progressive MS. Notably, MRI studies in progressive MS show less brain permeability, probably reflecting CNS compartmentalized inflammation. As a result, we have studied the effect of administering the oral multi-strain probiotic, Vivomixx, on the gut microbiome and plasma short chain fatty acids (SCFAs) in the late chronic phase of a murine model of PPMS, the Theiler’s murine encephalomyelitis virus induced demyelinating disease (TMEV-IDD).

## Results

### Vivomixx treatment on the symptomatic chronic phase of TMEV-IDD improves motor function

**Figure 1.**
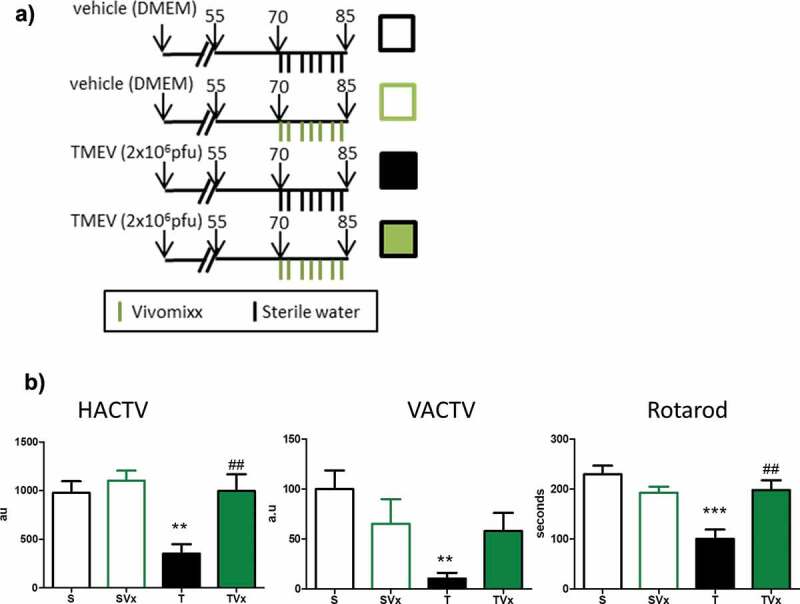
Vivomixx-treatment improves the motor disability of TMEV-mice. (a) Scheme of the experimental groups. Mice were administered 3 × 10^8^ cfu Vivomixx by oral gavage three times a week from day 70 pi to 85 dpi. Control mice were administered autoclaved water alone. b) Horizontal (HACTV) and vertical activity (VACTV), as well as motor coordination were evaluated. The data are presented as the means ± SEM: ***p < .001 vs. S; ##p < .01 vs T; n = 10 mice/group studied in two independent experiments.

We explored the effect of manipulating the gut microbiota by oral administration of Vivomixx during the chronic phase of disease (Figure 1(a)). Consumption of this probiotic improved motor function (Figure 1(b)), witnessed through the increase in the horizontal and vertical activity displayed by these mice (HACTV *p* < .01 *vs* TMEV-mice), as opposed to the more limited activity of the vehicle treated TMEV-mice (TMEV *p* < .01 *vs* sham mice). The reduced latency to fall observed in TMEV-mice on the rotarod performance confirmed the motor improvement induced by Vivomixx supplementation (*p* < .01 *vs* TMEV-mice).

### Effects of oral Vivomixx on gut microbiota and blood content of SCFAs

**Figure 2.**
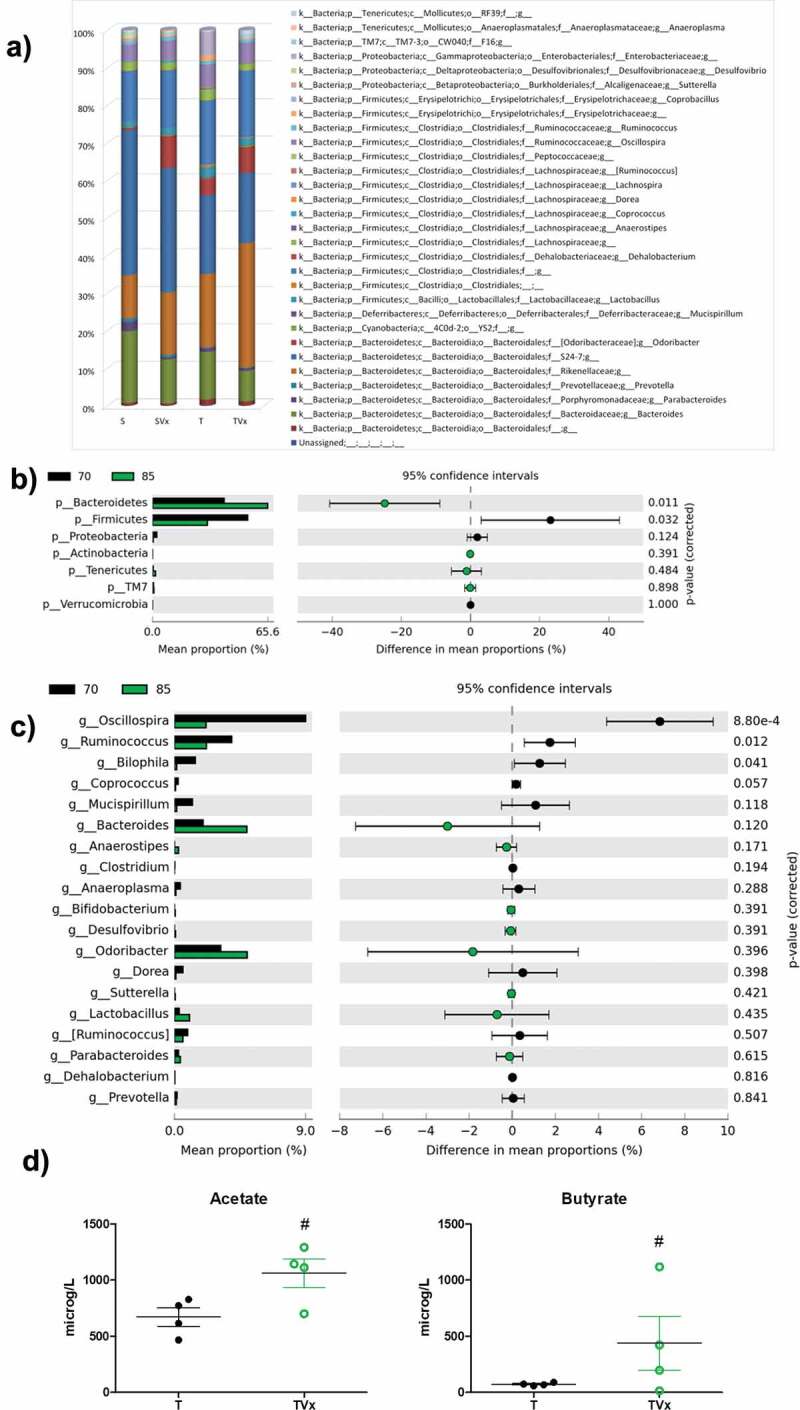
Changes in gut microbiota population after Vivomixx treatment. Fecal pellets were collected freshly from each mouse before and after probiotic treatment. A) Stacked bar charts of the relative abundance of main bacterial communities, at genus level, present in the different experimental groups. Extended error bar plots, at phylum (b) and genus level (c), shows the differences in the mean proportions of bacterial taxa in TMEV mice before (black) and after treatment (green). Corrected *p*-values are shown on the right. D) Quantification of the plasma levels of acetate and butyrate. The data are presented as the means ± SEM: ***p < .001 vs. S; ##p < .01 vs T; n = 5 mice/group.

Miseq Illumina analysis of the bacterial population revealed that TMEV-mice showed a decreased relative abundance of *Bacteroides, Parabacteroides, Prevotella, Sutterella* or *Desulfovibrio* while an increase of *Anaerostipes, Dorea, Erysipelotrichaceae* or *Coprobacillus* were observed (Figure 2(a)). Sham mice subjected to oral Vivomixx treatment experiment subtle changes in gut microbiota composition. In the case of Vivomixx-TMEV mice the relative abundance *Anaerostipes, Dorea, Oscillospira, Enterobacteraceae* or *Ruminococcus* were decreased while *Bacteroides, Odoribacter, Lactobacillus, or Sutterella* were increased (Figure 2(a)). To give robustness to our data we compare the gut microbiota composition of TMEV-mice before and after Vivomixx treatment, we found an increased relative abundance of *Bacteroidetes* and a significant decrease in *Firmicutes*. In addition, the percentage of bacteria belonging to *Actinobacteria, Tenericutes* and *TM7* phyla tend to increase, but not significantly (Figure 2(b)). At genus level (Figure 2(c)), Vivomixx treatment tend to rebalance microbiota changes increasing the relative abundance of *Bacteroides, Odoribacter* or *Lactobacillus*, while *Oscillospira, Ruminococcus* or *Bilophila* were significantly reduced. Gut microbiota changes would be related to the increased levels of acetate and butyrate observed in the plasma of TMEV-mice after 15 days of probiotic treatment (Figure 2(d)).

### Oral Vivomixx alters CNS infiltrates in the TMEV-IDD augmenting the frequency of the regulatory B220^+^CD19^+^CD5^+^CD1^high^ B lymphocytes

**Figure 3.**
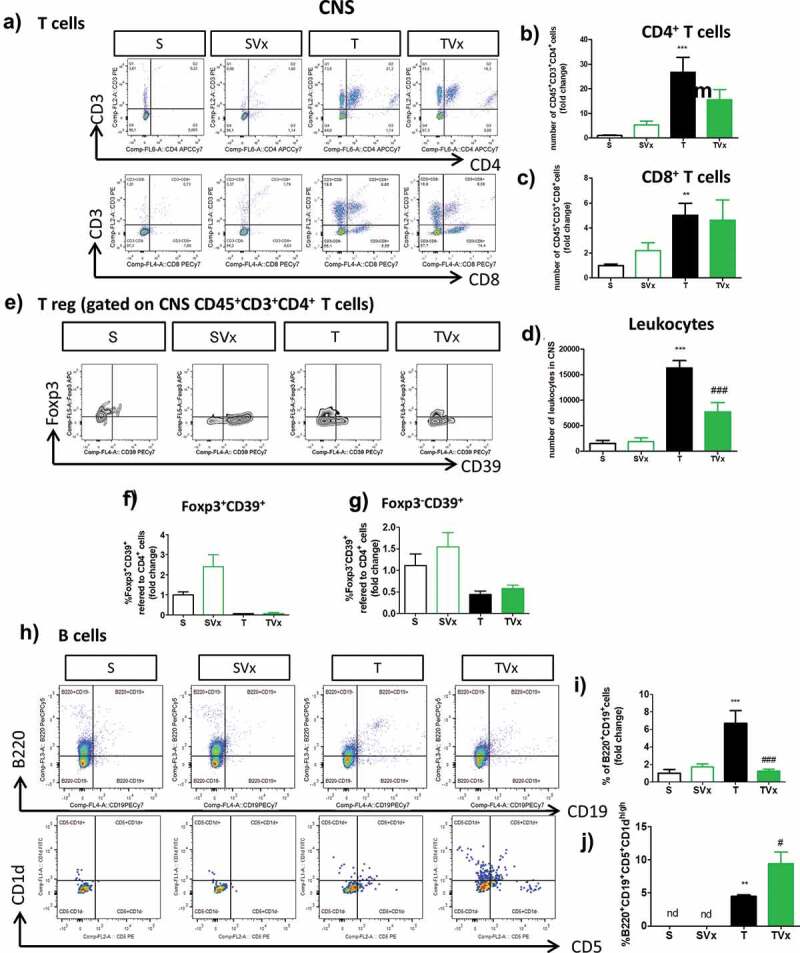
Effect of Vivomixx treatment on the T and B cell populations in the CNS. A single cell leukocyte suspension was obtained from the brain and spinal cord. A, H) Representative flow cytometry plots of CD45^+^CD3^+^CD4^+^ T cells, CD45^+^CD3^+^CD8^+^ T cells (a), and B220^+^CD19^+^ B cells and CD5^+^CD1d^high^ cells gated on B220^+^CD19^+^ B cells (h). B-D) Quantification of the change in the number of CD45^+^CD3^+^CD4^+^, CD45^+^CD3^+^CD8^+^ T cells and leukocytes relative to S mice E) Representative flow cytometry plots of CD39Foxp3 cells gated on CD45^+^CD3^+^CD4^+^ T cells. F-G) Percentage of Foxp3^+^CD39^+^ and Foxp3^−^CD39^+^ T cells gated on CD45^+^CD3^+^CD4^+^ T cells, respectively. The data represent the change relative to the S mice. I) Quantification of the change in the percentage of B220^+^CD19^+^ B cells relative to the S mice. J) Percentage of CD5^+^CD1d^high^ cells gated on B220^+^CD19^+^ B cells in the CNS: Groups S, T (n = 10); groups SVx, TVx (n = 5); **p < .01 vs S; ***p < .001 vs. S; #p < .05 vs. T; ###p < .001 vs T.

We assessed whether administering Vivomixx altered the T cell subpopulation in the CNS of TMEV-mice (Figure 3(a)). A tendency toward a reduction in the proportion of CD4^+^ T cells was observed (Figure 3(b)), with no change in the frequency of CD8^+^ T cells (Figure 3(c)). However, the increase in the number of leukocytes in the CNS of TMEV-infected mice was significantly dampened by probiotic supplementation (Figure 3(d)). In addition, probiotic gavage did not significantly modify the proportions of Foxp3^+^CD39^+^CD4^+^ or Foxp3^−^CD39^+^CD4^+^ T cells in the CNS of TMEV-infected mice (Figure 3(e-g)). Conversely, the presence of B cells (Figure 3(h)) was strongly influenced by oral Vivomixx administration, which significantly reduced the proportion of B cells (Figure 3(i); *p* < .001 TMEV *vs* sham; *p* < .001, TMEV plus Vivomixx *vs* TMEV). More interestingly, Vivomixx enhanced the presence of the Breg subpopulation B220^+^CD19^+^CD5^+^CD1d^high^ cells (Figure 3(j); *p* < .05 *vs* TMEV).

### Oral Vivomixx modifies microglial morphology and reduces astrogliosis promoting an anti-inflammatory profile in the spinal cord

**Figure 4.**
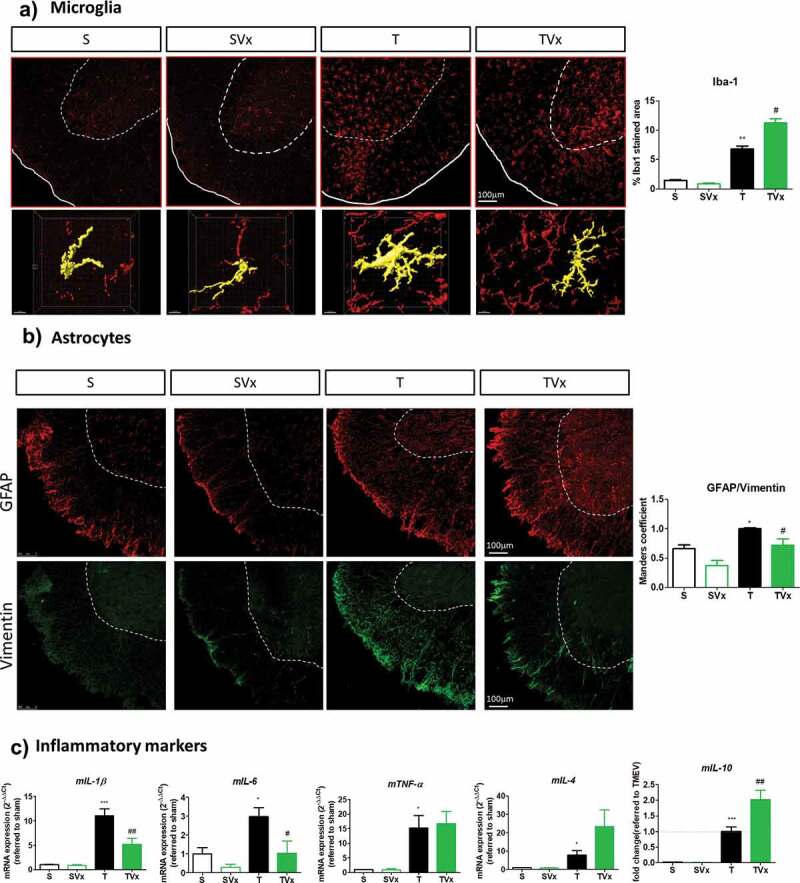
Vivomixx administration in the chronic phase of TMEV-IDD promotes anti-inflammatory responses in the spinal cord. A) Representative images of transversal spinal cord sections immunostained with Iba-1 to label microglia. Scale bar 100 μm. Below are higher magnification images (63x) of Iba-1 stained cells that indicate the changes in morphology. Scale bar 10 μm. Quantification of the area occupied by Iba-1 staining is also shown on the right. B) Representative images of GFAP (red) and Vimentin (green) staining in the spinal cord together the quantification of Vimentin-GFAP overlap through the Manders coefficient measure. C) qRT-PCR analysis of inflammatory markers (*mIL-1β, mIL-6, mTNF-α, mIL-4* or *mIL-10*) in the spinal cord as assessed using the 2^−ΔΔCt^ method. *p < .05 vs. S; **p < .01 vs. S; ***p < .001 vs. S; #p < .05 vs. T; ##p < .01 vs. T.

Microglial cells maintain the CNS homeostasis, being activated in MS and its experimental models. As expected, the area of Iba-1 staining is larger in TMEV-mice (*p* < .01 *vs* sham mice) and Vivomixx enhanced this effect, further increasing the area of Iba-1 staining (*p* < .05 *vs* TMEV: Figure 4(a)). Nevertheless, a detailed examination of these cells indicated subtle morphological changes in the spinal cord of mice that received Vivomixx reflecting that microglia exhibited anti-inflammatory activation or attenuated proinflammatory activity (Figure 4(a)). In addition, probiotic supplementation was associated with weaker Vimentin-GFAP co-localization in the spinal cord of TMEV-infected mice, whereas TMEV-mice had a higher MoC than sham mice (Figure 4(b), *p* < .05 *vs* sham mice and *p* < .05 *vs* TMEV-mice). Indeed, when we analyzed the expression of pro-inflammatory cytokine genes (Figure 4(b)), we found that the elevated expression of *IL-1β* and *IL-6* in TMEV-mice (*p* < .001 and *p* < .05 *vs* sham, respectively) was significantly reduced by Vivomixx (*p* < .01 and *p* < .05 *vs* TMEV-mice, respectively). Conversely, expression of the *IL-10* gene was promoted by Vivomixx (*p* < .01 *vs* TMEV-mice), while *IL-4* expression displayed a tendency toward enhancement. Intriguingly, the elevated *TNF-α* gene expression observed in TMEV-mice remained unchanged by the probiotic treatment (Figure 4(c)).

### Peripheral immunological changes in the symptomatic chronic phase of TMEV-IDD associated with Vivomixx supplementation

**Figure 5.**
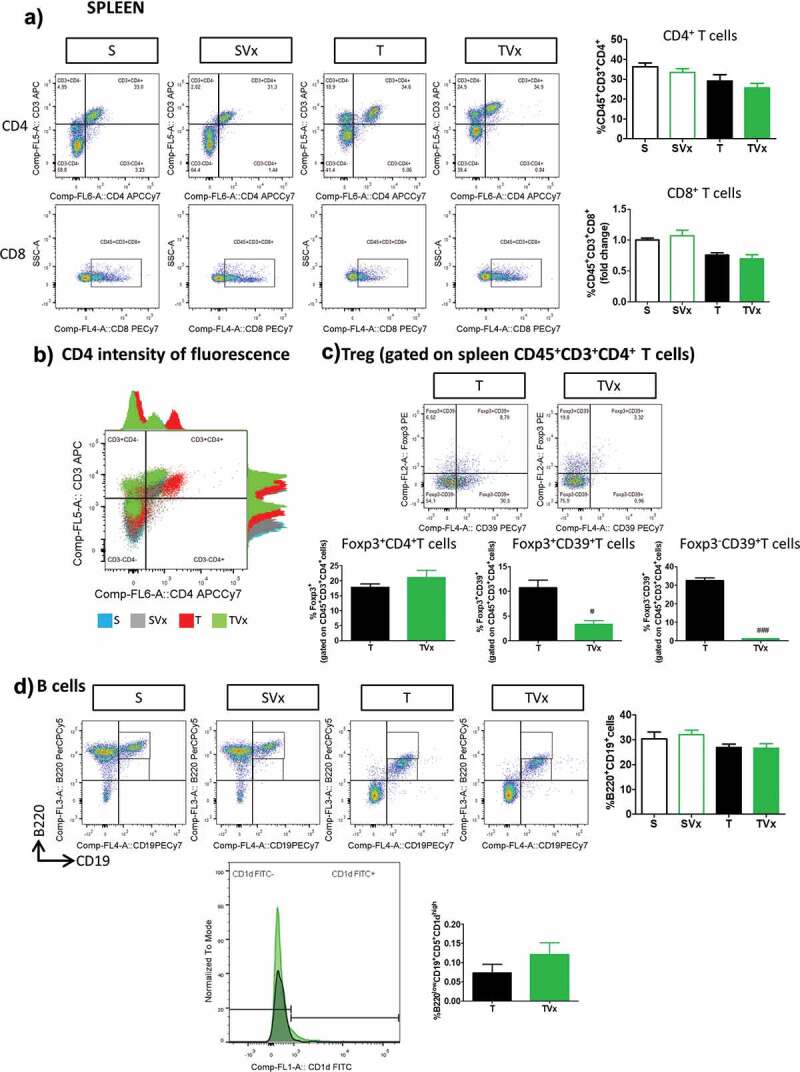
Subtle changes in the peripheral immunological response after Vivomixx administration to TMEV-mice. A spleen cell suspension was stained to analyze the CD4, CD8, CD39^+^ Treg and Breg cell populations. a) Representative flow cytometry plots of CD45^+^CD3^+^CD4^+^ T cells and CD45^+^CD3^+^CD8^+^ T cells, along with their corresponding quantification. b) Histogram of fluorescence emitted by CD3^+^CD4^+^ spleen cells from representative mice in each experimental group. c) Flow cytometry plots of Foxp3^+^CD39^+^ Treg cells gated on CD45^+^CD3^+^CD4^+^ T cells, together with a quantification of the percentage of Foxp3^+^ T cells (gated on CD4^+^ T cells), Foxp3^−^CD39^+^ and Foxp3^+^CD39^+^ Treg cells. d) Representative flow cytometry images and quantification of the B220^+^CD19^+^ cells in the spleen, together with a normalized histogram and quantification of CD1d expression in B220^+^CD19^+^CD5^+^ gated cells. Groups. S, T (n = 10); groups SVx, TVx (n = 5); **p < .01 vs S; ***p < .001 vs. S; #p < .05 vs. T; ###p < .001 vs T.

The proportions of CD4^+^ and CD8^+^ T cells in the spleen of TMEV-mice were not significantly affected by Vivomixx administration (Figure 5(a)), although more intense CD4^+^ fluorescence was evident in T cells from TMEV-mice and this was dampened when they received the probiotic (Figure 5(b)). Although the frequency of Treg cells was similar in TMEV-mice irrespective of their treatment with Vivomixx, the percentage of Foxp3^+^CD39^+^ and Foxp3^−^CD39^+^ T cells was dramatically reduced in the spleen of TMEV-mice that received Vivomixx (*p* < .05 and *p* < .001 *vs* TMEV, respectively) (Figure 5(c)). By contrast, there were no significant changes in the proportion of B cells (Figure 5(d)) or in the abundance of Breg cells in the spleen of TMEV-mice receiving Vivomixx.

**Figure 6.**
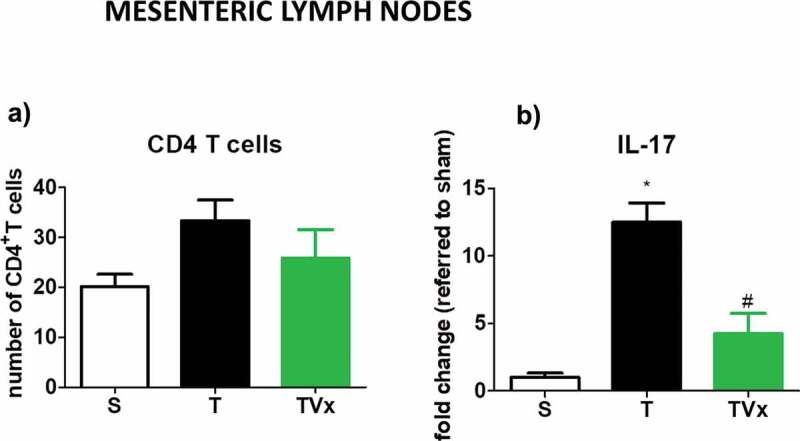
Vivomixx affects the Th17 responses in the MLNs. CD4 T cells isolated from MLN were polarized to Th17 in culture. The expression of IL-17 was measured by ELISA, and the data represent the mean ± SEM of the change relative to the S or T mice from two experiments performed in triplicate: *p < .001 *vs* S; #p < .05 *vs* T.

No significant variation was detected in the number of CD4^+^ T cells in the MLNs under the different conditions studied (Figure 6(a)). However, the release of IL-17 by Th17 polarized lymphocytes derived from TMEV-mice was stronger respect to sham mice (*p* < .05) (Figure 6(b)). By contrast, significantly less IL-17 was produced by CD4^+^ T cells obtained from the MLNs of TMEV-mice that received Vivomixx (*p* < .05 *vs* TMEV-mice).

## Discussion

In the last decade, evidence has emerged of a relationship between gut microbiota and MS, both in patients and in animal models.^4,5,18,19^ In addition, there is significant support for the gut microbiota playing a critical role in shaping the immune system.^20–22^ Several reports described, in EAE models, a therapeutic effect of multi-strain probiotics^15^ and specific bacterial strains like *Bacteroides*,^9,10^ *Prevotella*^11^ or *Lactobacillus*. However, the involved mechanisms for their benefits seem to be different. For example, while *L. paracasei* DSM13434 and *L. plantarum* DSM 15312 increased TGF-β1 in the blood, *L. plantarum* DSM 15313 enhanced blood levels of IL-27.^12^ Until date the only probiotic considered a kind of medical food is Vivomixx^23^.Here, we proved the efficacy of Vivomixx treatment in a model of progressive MS whereby oral administration of this probiotic, during the chronic phase improved the motor disability likely associated to changes in gut microbiota.

Differences in the composition of the fecal microbiota at the genus level have been reported in a variety of studies on MS patients.^4,5,18,19^ In our study TMEV-mice showed changes in gut microbiota population compatible with MS disease like decreases of *Prevotella, Bacteroides, Parabacteroides or Coprobacillus* relative abundance or an increase of *Anaerostipes*.^18,24,25^ Vivomixx treatment tends to decrease *Anaerostipes, Dorea, Oscillospira, Enterobacteraceae* or *Ruminococcus* while *Bacteroides, Odoribacter, Lactobacillus, or Sutterella* were increased. These changes would be associated with the beneficial effects of probiotic treatment. For example, *Dorea* have been considered to be part of the healthy gut microbiota or patients on disease-modifying treatment show increased abundance of *Sutterella and Prevotella*.^5^

In the TMEV-IDD model we found that mice receiving oral Vivomixx had fewer CNS infiltrated leukocytes, particularly B cells, without significant changes in the proportion of CD4^+^ and CD8^+^ T cells, Treg cells, or Foxp3^+^CD39^+^ T cells. One of the pathways by which the commensal microbiome affects host immunity at distal sites is by regulating the activity of Treg cells through the ectonucleotidase CD39.^10^ Particularly, CD39^+^ cells have been associated with the suppression of neuroinflammation by *Bacteroides fragilis* in the EAE model^26^ and its polysacharide A was not able to protect against EAE in CD39-deficient mice.^10^ Here, TMEV infection decreased CD39 Treg cells population in the CNS, in accordance with our previous results^27^ and previous data from the peripheral blood of MS patients.^28^ Unlike what we might expect, probiotic treatment did not reverse the decreased CD39^+^ Tregs suggesting that CD39 activity is not the main pathway involved in the anti-inflammatory effects of Vivomixx. Indeed, our results are consistent with data from MS patients who did not show changes in the frequencies of CD4, CD8 or CD39^+^T cells following Vivomixx.^17^

It is known that in EAE, encephalitogenic T cells upregulate their CD4 cell surface expression following antigen recognition and that this was associated with EAE development, for example after transfer to naïve animals.^29^ Therefore, although oral Vivomixx did not affect the frequency of CD4^+^ T cells, the fact that this probiotic restricted the surface expression of CD4 on T cells suggest its possible contribution to its beneficial effects in TMEV mice.

Recent reports show that gut microbiota and microbial products can directly regulate B-cell development, activation, and differentiation.^30^ In addition, the induction of Breg cells in an autoimmune context was seen to depend on a healthy microbiota.^31^ Notably, in our model of progressive MS, Vivomixx increased the frequency of Breg cells, unveiling a different mechanism of action to that exerted by other probiotics in EAE models where Treg cells have been specifically implicated.^26,32^ Nonetheless, Bregs would be involved in the beneficial effect of Vivomixx but functional studies are needed to be sure that these cells are working in an effective way. There are many crucial differences between EAE and the TMEV model. First, the TMEV ability to maintain the inflammation into the CNS compartment, with minor effects in the periphery unlike that observed in EAE. On this way, most of studies noticed the induction of IL-10 producing Tregs in the periphery as the main mechanism for EAE protection when mice received many probiotics as *Lactobacilli*^12^ or *Prevotella Histicola*.^11^ The second possibility comes from the different strain of mice used in the models of MS. In EAE models that use SJL/L mice, like we used in the present study, the protective effects against EAE following *Lactobacillus helveticus* seems to involve decreased IL-6 and reduced Th-17 cells in the spinal cord, without changes in Tregs.^14^

Preclinical studies suggest that the microbiome plays a critical role in regulating microglial maturation and function.^33^ Indeed, microglia from TMEV-infected mice administered Vivomixx undergo morphological changes, which could reflect changes in their functional state, in accordance with the emergent hypothesis that microglia may adopt distinct CNS region-specific phenotypes.^34^ In the spinal cord, the pro-inflammatory environment of TMEV-mice shifted to an anti-inflammatory profile following Vivomixx supplementation.

In an attempt to find a mechanism that may explain the changes in the CNS of TMEV-mice that receive oral Vivomixx we assessed plasma acetate and butyrate. On the whole, it seems that the reduction in bacteria producing SCFAs may be a common characteristic of MS patients.^18^ A high fiber diet or SCFA supplementation are reported to ameliorate EAE^35^ and, there is growing evidence supporting that circulating metabolites of the microbiota can influence CNS and immune cell activity,^33^ including microglial function.^27,36^ One of the most significant findings of the present study was the increased butyrate and acetate levels in the plasma of TMEV-mice that received Vivomixx which may contribute to the altered immune CNS responses and the improved motor activity. Indeed, several reports indicate the beneficial effects of SCFA suppressing inflammation in the EAE model.^35,37,38^ Furthermore, a recent finding shows that specific SCFAs exert an epigenetic modulation of Breg responses.^39,40^ Particularly, EAE mice that were subjected to the transference of Breg cells, expanded *in vitro* in presence of pentanoate, did not develop the classical symptoms. Furthermore, butyrate increased the levels of 5-Hydroxyindole-3-acetic acid, a metabolite of serotonine neurotransmitter which activates the aryl-hydrocarbon receptor (AhR), a newly discovered transcriptional marker for Breg function.^41^ Here, we speculate that Breg response as well as the reduction of IL17 production by Th17 mesenteric polarized cells may be associated with acetate and butyrate increments following Vivomixx administration to TMEV-mice.

Although, there is no clear evidence of a relationship between SCFA and Bregs at CNS level, astrocytes express AhR that, when activated limit CNS inflammation. In the EAE model the results by Quintana’s group^42^ suggest a link between metabolites derived from dietary tryptophan by the commensal gut bacteria and the activation of AhR signaling in astrocytes. It is worth noting that our understanding of the molecular pathways involved in the effects of probiotics and SCFAs on CNS inflammation, in MS and its animal models, is still very limited. This statement also includes microglial cells which are thought to be targeted by SCFAs,^36^ although their underlying mechanisms remain incomplete too. On this line, more recently, a new pathway in which microbial metabolites may limit the pathogenic activity of microglia and astrocytes has been identified.^43^ Then, we would like to highlight the reduction of astrogliosis and microgliosis we found in Vivomixx treated-TMEV mice.

An important issue to consider is that MS disease modifying therapies may alter the microbiome directly. As an interesting example, the abundance of *Prevotella* was increased in MS patients treated with glatiramer acetate taking into account that MS reduced the frequency of these bacteria.^44^ Furthermore, in a humanized transgenic EAE preclinic model of MS, the combination of human *Prevotella histicola* and glatiramer acetate did not have a synergistic effect, but *Prevotella* was as effective as glatiramer in suppressing the disease.^11^

While dogma dictates that Firmicutes are responsible for butyrate and Bacteroidetes are responsible for acetate and propionate production, there is evidence for alternative pathways.^45^ There are non-canonical butyrate-producing pathways that use amino acids as primary substrates, including the lysine, 4-aminiobutyrate, pathways. Considering metagenomic analysis of samples from healthy human patients from the Human Microbiome Project a significant overlap between the genes involved in the lysine and 4-aminobutyrate pathways and those expressed by members of the phylum Bacteroidetes was found for butyrate production. Then, it was predicted that these both pathways can contribute to the butyrogenic potential of Bacteroidetes.^46^ Because members of the *Odoribacter* genus dominated the lysine and 4-aminobutyrate pathways, our finding about increased *Odoribacter* abundance would be associated with the increased levels of butyrate in TMEV-mice treated with Vivomixx.

The subtle changes promoted by Vivomixx at spleen level are in accordance with the compartmentalization of inflammation that occurs in progressive MS, characterized by intrinsic CNS processes driven by resident cells like microglia and astrocytes. As such, the main immunological changes in the chronic phase of TMEV-IDD linked to the manipulation of the gut microbiota by oral Vivomixx happen within the CNS. Although probiotics do not modify the host microbiome in a robust and persistent manner, they can influence gut immunity and homeostasis.^47^ Such effects were evident in the TMEV model through the CNS immune responses, microgliosis and astrogliosis, IL-17 production by *ex vivo* mesenteric CD4^+^ T cells, and more importantly, the severity of motor dysfunction.

Regarding probiotics as therapeutic agents, VSL#3 administered to a small number of RRMS patients leads to an anti-inflammatory peripheral immune response.^17^ It seems clear that both, disease promotion and amelioration may be induced by specific components of the gut microbiota that interact bi-directionally with the host immune system. This concept implies that selective experimental strategies manipulating specific subsets of bacteria or balancing specific gut microbiota may be useful as a therapeutic approach in MS. Our findings may provide a starting point for further mechanistic studies that help to develop adjuvant treatments complementary to the available therapeutic options for progressive MS patients.

## Materials and Methods

### Animals and TMEV infection

TMEV-infected susceptible female SJL/J mice (Charles River) were maintained under conventional controlled conditions. The right brain hemisphere of 6–8 weeks-old mice was inoculated with 2 × 10^6^ plaque forming units (pfu) of the Daniel (DA) strain of TMEV). Sham control mice received the vehicle alone. Each experimental group was housed in different cages and in different isolated racks in the same room. Each cage contained 5 mice subjected to the same treatment, receiving the same food and sterile water, and manipulated by the same person. All experiments were performed in strict accordance with EU (Directive 2010/63/EU) and Spanish regulations (Royal Decree 53/2013 BOE n° 34 and “Comunidad de Madrid” decree: ES 280790000184). The Ethics Committee on Animal Experimentation at the Instituto Cajal approved all the procedures employed in this study (protocol number: 2013/03 CEEA-IC).

### Treatments

TMEV-mice were orally gavaged with 3 × 10^8^ cfu (100 μl) of Vivomixx for 70 to 85 days post infection (dpi) (TVx group; n = 10). Vivomixx, a multi-species probiotic is composed of *Lactobacillus paracasei* DSM 24734, *Lactobacillus plantarum* DSM 24730, *Lactobacillus acidophilus* DSM 24735, *Lactobacillus delbruckeii subspecies bulgaricus* DSM 24734, *Bifidobacterium longum* DSM 24736, *Bifidobacterium infantis* DSM 24737, *Bifidobacterium breve* 24732 and *Streptococcus thermophilus* DSM 24731. Sham or TMEV-mice administered the vehicle (S group, n = 10 or T group, n = 10, respectively) and Sham mice administered Vivomixx (SVx group, n = 5).

### Evaluation of motor function

Mice were followed for the progression of demyelinating disease by evaluating spontaneous motor activity on day 70 and 85 pi using an activity cage (Activity Monitor System Omnitech Electronics, Inc.; Colombus, OH, USA) and motor coordination by rotarod test as we previously described.^48^

### Sample collection

Fecal samples freshly obtained from each mouse on day 70 and 85 pi were stored at −80°C. On day 85 pi, spleen and MLN were removed from anesthetized mice (Dolethal; 50 mg/kg body weight) for flow cytometry and *ex vivo* CD4-T cell studies. Then, anesthetized mice were perfused transcardially with saline to obtain the brain and spinal cord for further FACS analysis.

### Metabolite analysis

Blood samples in EDTA-treated BD vacutainer were centrifuged for 10 min at 1500 g in a refrigerated centrifuge within two hours of extraction and aliquots were stored at −20°C. Acetate and butyrate levels were assessed as described previously.^27^ Validation of the method was carried out using^13^C-acetate and^13^C-butyrate standards. For both analytes the recoveries were above 90% and the precision, in terms of relative standard deviation, was below 10%.

### *E*x vivo *experiments on CD4 T cells*

CD4^+^ T cells were isolated from the MLN using a commercial cell isolation kit and following the manufacturer’s instructions (Miltenyi, Biotec Inc). Cells from each animal were cultured and polarized to Th17 cells in RPMI supplemented with: 2-β Mercaptoethanol (50 μM); immobilized anti-mouse CD3ε; anti-mouse CD28; anti-mouse IFN-γ; anti-mouse IL-4 (all from BD biosciences); hTGF-β and recombinant murine IL-6 (from Peprotech); and recombinant murine IL-23 (BioLegend). The cells were stimulated 3 days later for 5 hours with PMA (100 ng/ml) and ionomycin (500 ng/ml, both from Tocris Bioscience). The supernatants were collected and stored at −20°C for IL-17 measurement by solid phase sandwich ELISA using Quantikine kits (M1700; R&D systems Inc), according to the manufacturer’s instructions. The assay’s sensitivity was 5 pg/ml.

### Tissue processing and immunohistochemistry

Anesthetized mice were perfused transcardially with saline and the spinal cord processed as previously described.^48^ Microglia were labeled with a primary antibody against Iba-1 (ionized calcium binding adaptor molecule 1, 1:1,000: Wako Chemical Pure Industry), while reactive astrogliosis was assessed by GFAP (1:2000: Sigma) and Vimentin (1:1000: Sigma) labeling. Five spinal cord sections were examined per animal and at least three mice per group were used to quantify the immunostained area using ImageJ software (NIH, Bethesda). The white matter area in the ventral and lateral horns was analyzed by immunofluorescence on a Leica TC SP5 confocal microscope. Representative images of the surface of a single microglial cell were obtained with Imaris software. Astroglial activation defined as the amount of Vimentin relative to the GFAP staining (Vimentin-GFAP overlapping) was analyzed using the Manders overlap coefficient (MoC) that quantifies the degree of co-localization of the two fluorophores.

### RNA extraction, reverse transcription and RT-PCR

Total RNA from the cervical spinal cord was extracted and then transcribed into cDNA as we previously described.^49^ Real-time PCR (RT-PCR) was performed with SYBR® and the following oligonucleotide primers *mIL-1*β, forward 5ʹ-TGG TGT GTG ACG TTC CCA TT-3ʹ, reverse 5ʹ-TCC ATT GAG GTG GAG AGC TTT C-3ʹ; *mTNF-α*, forward 5ʹ-AGA GGC ACT CCC CCA AAA GA-3ʹ, reverse 5ʹ-CGA TCA CCC CGA AGT TCA GT-3ʹ; *mIL-4*, forward, 5ʹ-CCACGGATGCGACAAAAATC-3ʹ, reverse 5ʹ-GACGTTTGGCACATCCATCTC-3ʹ; *mIL-10*, forward 5ʹ-TGA ATT CCC TGG GTG AGA AGC TGA-3ʹ, reverse 5ʹ-TGG CCT TGT AGA CAC CTT GGT CTT-3ʹ; *mIL-6*, forward 5ʹ-TCC AGA AAC CGC TAT GAA GTT C-3ʹ, reverse 5ʹ-CAC CAG CAT CAG TCC CAA GA-3ʹ; and *mRps29* forward 5ʹ-GCC GCG TCT GCT CCA A-3ʹ, reverse 5ʹ-ACA TGT TCA GCC CGT ATT TGC-3ʹ. PCR amplification were initiated incubating at 50°C for 2 min and 95°C for 10 min, and amplification was performed over 40 cycles at 95°C for 15 s and 60°C for 1 min. The cDNA samples were analyzed in triplicate on an Applied Biosystems PRISM 7500 Sequence detection system, normalizing the mRNA expression to the *Rps29* gene in each sample and quantifying gene expression by the 2^−ΔΔCt^ method. The results are expressed relative to the Sham mice for each time point.

### Flow cytometry antibodies

A CNS or spleen leukocyte suspension was obtained as described previously.^49^ Freshly isolated cells (10^6^) were incubated with an anti-mouse CD16/CD32 FC (eBioscience) and labeled with anti-mouse antibodies: PE anti-CD25; PE anti-CD3; PerCP-Cy5.5-conjugated anti-CD45; PerCP-Cy5.5-conjugated anti-B220; PE-Cy7-conjugated anti-CD39; PE-Cy7-conjugated anti-CD8a; PE-Cy7-conjugated anti-CD19; APC anti-CD3; APC-Cy7-conjugated anti-CD4; APC-Cy7-conjugated anti-CD11b (all from BD Pharmingen); APC anti-CD3; and PE-Cy7-conjugated anti-CD39 (both from eBioscience). To detect Foxp3, the cells were suspended in the fixation/permeabilization buffer and stained with a PE anti-Foxp3 antibody (BD Pharmingen). Cell viability was assessed with the LIVE/DEAD Fixable Green Dead Cell Stain Kit or LIVE/DEAD Fixable Red Dead Cell Stain Kit (Life Technologies Corporation). At least 10,000 events were acquired in each experiment on a FACSaria flow cytometer (BD Biosciences) and the data were analyzed using FlowJo software (v.10; Tree Star).

### 16S rRNA analysis of the microbial community

Fecal DNA was isolated using the QIAamp DNA Stool Mini Kit (Qiagen; cat. no. 51504) and following the stool pathogen detection protocol. DNA was extracted following the manufacturer’s instructions with the modifications described previously,^27^ and the quality of the reads was analyzed with FastQC software (http://www.bioinformatics.babraham.ac.uk/projects/fastqc/). The microbiome analysis of the paired-end reads was performed using the QIIME2 v2018.8 software (Qualitative Insights into Microbial Ecology)^50^ and the sequences were grouped as “sequence variants” (SVs), equivalent to the 100% Operational Taxonomic Units (OTUs). Representative sequences from each cluster were queried against the GreenGenes database (version 13_8) to obtain taxonomic assignments at a 97% sequence identity using BLAST. These representative sequences were then used for phylogenetic analysis and for diversity calculations.

### Statistical analysis

The data were expressed as the mean ± standard error or the mean (SEM) and they were analyzed using GraphPath Prism5 Software. One-Way ANOVA followed by the Bonferroni *post hoc* test were used to determine the statistical significance. For non-parametric analyzes, a Kruskall-Wallis and Mann-Whitney U test were applied.
